# Spinal Injuries from Equestrian Activity: A US Nationwide Study

**DOI:** 10.3390/jcm14134521

**Published:** 2025-06-26

**Authors:** Randall T. Loder, Alyssa L. Walker, Laurel C. Blakemore

**Affiliations:** 1 Riley Hospital for Children, Indiana University School of Medicine, Indianapolis, IN 46202, USA; 2 Department of Orthopaedic Surgery, George Washington University School of Medicine, Washington, DC 22031, USA

**Keywords:** spine, equestrian, fracture, emergency department, demographics

## Abstract

**Background/Objectives:** Equestrian activities can result in spine injuries. Most studies are from single centers, and none use a national database. It was the purpose of this study to describe the demographics, injury mechanisms, and types of equestrian-associated spinal injuries using a US national ED database. **Methods:** The National Electronic Injury Surveillance System database was queried for equestrian-related spine injuries from 2000–2023. ED disposition was categorized as discharged or not discharged. Statistical analyses accounted for the weighted, stratified nature of the data to obtain national estimates. **Results:** There were an estimated 54,830 patients, having an average age of 42 years. Most were female (73.6%) and White (93.7%); one-half (51.1%) were not discharged from the ED. The spine level was the lumbar (49.1%), thoracic (24.4%), sacrococcygeal (15.5%), and cervical (11.0%) spine. Multiple spine fractures occurred in 4.0%. A simple fall off a horse occurred in 53.6% of the injuries, and the patient was bucked/thrown/kicked off the horse in 39.7%. Neurologic injury was rare (1.8%). Hospital admission was highest in the cervical group (74.3%) and lowest in the sacrococcygeal group (33.5%). The cervical group had the highest percentage of males (43.7%) compared to the thoracic, lumbar, and sacrococcygeal groups (22.8%, 27.3%, 16.8%, respectively). There were proportionally fewer females in those over 50 years of age, where the male percentage was 11.7%, 25.6%, and 31.6% for those <18 years, 18–50 years, and >50 years old, respectively. **Conclusions:** This large study can be used as baseline data to evaluate further changes in equestrian injuries, especially the impact of further prevention strategies, education protocols, and legislative/governmental regulations of public equestrian localities.

## 1. Introduction

Equestrian involvement with humans can lead to injury [1] involving the spine (fractures and/or spinal cord injuries) [2,3,4,5,6,7,8,9,10,11,12,13,14,15,16]. Some may be catastrophic, as demonstrated by the cervical spine C1/2 fracture sustained in 1995 by the actor Christopher Reeves (who played “Superman”), which left him ventilator-dependent [17,18].

There are some studies specifically investigating equestrian-associated spine injuries [3,19,20,21,22]. However, most of these are single-center studies with relatively small numbers, come solely from trauma centers, do not encompass an entire nation, or only study those admitted to the hospital. None use an entire national database for those both discharged from an emergency department (ED) and admitted to the hospital, both non-trauma and trauma centers, and across all age groups (pediatric and adult). It was our purpose to describe the demographics, injury mechanism, and types of spinal injuries sustained by humans from equine activity using a US national ED database, including both patients discharged and admitted from the ED. This information will give a “high-altitude” view of the problem and can be used for assessing the outcomes of future prevention strategies, assisting hospital administrators in resource allocation, and providing detailed knowledge to health care providers taking care of patients with spinal-associated injuries due to equestrian activity.

## 2. Materials and Methods

The data from the National Electronic Injury Surveillance System (NEISS) database was used for this study. The NEISS prospectively gathers data on a daily basis due to injuries from ~100 hospitals in the United States and its territories having an ED. NEISS data is based on a nationally representative probability sample of hospitals in the U.S. and its territories that have at least 6 beds and an ED. It is operated by the US Consumer Product Safety Commission. The sample is stratified based on ED size (number of annual ED visits) and geographic location. These 100 hospitals are grouped into five strata. The strata are denoted as small [0–16,830], medium [16,831–21,850], large [28,151–41,130], and very large [>41,130], and one consisting of children’s hospitals of all sizes. Each hospital is given a weight which is equal to the inverse of the probability of selection for the hospitals in each stratum. Geographically, the hospitals are very diverse and cover the entire US. (In 2018, using the NEISS online map (https://www.cpsc.gov/s3fs-public/NEISS_Hospital_Map_2018.pdf?6gAfTlFla.YEZWTkBH5hF6zcHm.1eweZ; accessed on 23 June 2025), it was noticed that there were 95 hospitals that came from 43 states and Puerto Rico; there were none from Nevada, New Mexico, Kentucky, Rhode Island, Maine, Hawaii, and Alaska. There were 47 small, 10 medium, 6 large, 24 very large, and 8 children’s hospitals.) Thus, due to its design, the NEISS data is nationally representative. From this stratified and weighted data, an estimated total number of injuries treated in hospital EDs is calculated, giving a statistically valid national estimate.

The database includes standard demographic variables, as well as a narrative column that gives a clinical vignette of each patient. The NEISS data is in the public domain and can be found online at https://www.cpsc.gov/cgibin/NEISSQuery/home.aspx (accessed on 23 June 2025). Similarly, acquisition/guidelines for its use can be obtained at www.cpsc.gov/library/neiss.html (accessed on 23 June 2025). This study was determined to be exempt by our local Institutional Review Board as the data is in the public domain.

This study was modeled after a previous study from the same authors [23], except that the search criteria were different due to a separate subject matter/research question. The NEISS data for the consumer product code of 1239 (horseback riding—activity, apparel, or equipment) was downloaded for the years 2000–2023. From this group those with a diagnosis code of 57 (fracture) or 61 (nerve injury) were extracted for the body parts of 89 (neck), 31 (upper trunk), and 79 (lower trunk). Until recently NEISS coders only entered the most severe diagnosis; thus, the narrative comments were reviewed using the Excel command FIND for other phrases to identify spinal injuries. These searches were performed since a patient with a more severe injury (e.g., pneumothorax, splenic injury, or brain hemorrhage) could also have sustained a spinal injury but would not have been coded as such due to the other injury being the more severe one. First, each vertebral level from C1 to L5 was searched using those terms (e.g., C1, C2, C3, etc.), along with “spine, vert, cerv, thor, lumb, occi, cond, odont, burst, compr, Jefferson, hangm, sac, cocc, and tailb” to find as many vertebral locations as possible, with sac used to find sacral injuries and cocc for coccygeal injuries. Next, the terms “trans, TP, TV, process, and SP” were used to find transverse process and spinous process fractures. Neurologic injuries were searched for using the terms “para, quad, compr, pleg, and cord”. The narrative comments for the patients uncovered with said search were reviewed, and when it was clear that it was a spine injury, then it was incorporated with those from the NEISS codes of 57 and 61. This comprised the final data set. Disposition from the ED was categorized as discharged or not discharged; patients transferred from the initial NEISS hospital to another facility were defined as not discharged. Race was classified as White, Black, and other as per the NEISS.

Using the narrative comments, five groups regarding the mechanism of injury were created: (1) was the patient bucked, thrown, or kicked off of the horse; (2) did the patient get kicked by the horse; (3) was the patient mounting the horse, mounted on the horse, dismounting from the horse, or not on the horse; (4) was the horse spooked; and (5) did it occur during an intentional jumping activity. We also ascertained if the tack (saddle, bridle, and related parts of equipment worn by the horse) was specifically involved or thought to cause the injury.

Two different age groups were created. The first grouping was those <16 and ≥16 years of age, as this cut off reflects when the skeleton typically becomes mature, with the known differences in fracture patterns between skeletally immature and skeletally mature individuals [24,25]. The second grouping was those <18 years, 18–50 years, and >50 years of age. These groups reflect minors who have not yet achieved legal adult status, adults, and older adults. The age limit of 50 years for older adults was selected as it is known that equestrians in North America are predominantly female [26,27,28], and 50 years of age is the average age of female menopause [29]. The menopause limit was chosen as osteopenia/osteoporosis becomes more prevalent in post-menopausal women [29] and might impact the prevalence and/or patterns of spine fractures between women of different ages.

The narrative comments were reviewed for each fracture to find which vertebra/vertebrae was/were involved for the cervical, thoracic, and lumbar spine. The most proximal vertebra was used to classify the fracture as cervical, thoracic, lumbar, or sacrococcygeal spine. The total number of fractures was tabulated. Finally, alcohol involvement was determined as in previous studies [30,31] by searching for the keywords BAC (an acronym for blood alcohol involvement), alcohol, EtOH, intoxicated, drinking, drank, drunk, club, ethanol, saloon, tavern, liquor, booze, beer, whiskey, brandy, rum, vodka, scotch, tequila, wine, sake, champagne, and cognac.

### Statistical Analysis

As the NEISS data set comprises only ~100 hospitals, the size of the hospital, categorized by the annual number of ED visits, was used to weight and stratify the data. With such stratified and weighted data, statistical analyses need to account for such a design [32] and obtain national estimates and 95% confidence intervals of the estimate (given in brackets). When the actual number of patients (n) is < 20, the estimated number (N) becomes unstable and should be interpreted with caution; thus, we reported both the n and N [33]. While the overall number of patients and major subgroups was large, when breaking down by several subgroups, the number of patients became small for certain subgroups. Therefore, both weighted and non-weighted analyses were performed, using the non-weighted sample simply as a cohort of patients in a traditional, retrospective manner of prospectively collected data. Continuous data are expressed as the average ± 1 standard deviation, and categorical data as frequencies and percentages. Differences between continuous variables were analyzed with the Student *t*-test and ANOVA. Differences between categorical variables were analyzed with Fisher’s exact test for 2 × 2 tables and the χ^2^ test for tables larger than 2 × 2. For the weighted analyses the SUDAAN 11.0.01™ software (RTI International, Research Triangle Park, NC, USA, 2013) was used. For non-weighted analyses the Systat 13.1 software (Palo Alto, CA, USA, 2009) was used. For all the analyses a *p* < 0.05 was considered statistically significant.

## 3. Results

### 3.1. General Demographics

**Figure 1:**
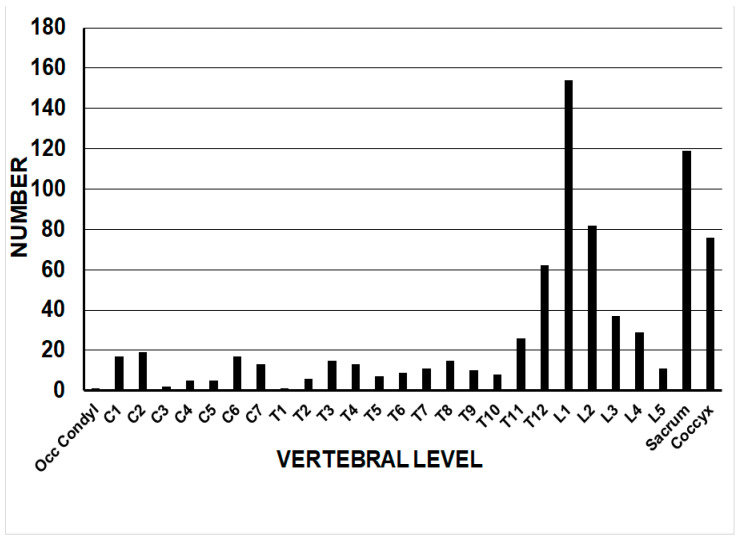
The location of the most proximal spine fracture in 770 patients due to horse-related injuries.

There were 34,091 actual patients seen in the EDs for equestrian-related injuries, for an estimated 1,462,193 [1,314,365–1,611,021]. There were 1294 actual ED visits for spine injuries over the 24-year period of 2000–2023, or an estimated 54,830 [46,233–63,427]. Thus, spine injuries account for 3.75% of all equestrian-related ED visits (54,830 out of 1,462,193). The average age was 42.4 [41.2, 43.7] years, the median age was 43.7, and the interquartiles [28.5, 54.9] (Table 1). The majority were female (73.6%) and White (93.7%); one-half (51.1%) were not discharged from the ED. A fracture occurred in 99.9% of the cases. The anatomic level of the fracture was lumbar (49.1%), thoracic (24.4%), sacrococcygeal (15.5%), and cervical (11.0%). Using non-weighted data, the anatomic distribution of spine fractures showed a predominance at the thoracolumbar junction, with 42.1% involving T12-L2 (Figure 1). There were multiple-level fractures in 4.0%, and an internal organ injury was present in 5.7%. A simple fall off the horse occurred in 53.6%, and the patient was bucked/thrown/kicked off the horse in 39.7%. The majority were seen at small hospitals (38.2%). Neurologic injury was rare (1.8%). Alcohol involvement was present in 1.7% of the patients.

### 3.2. Differences by Spine Levels

**Figure 2:**
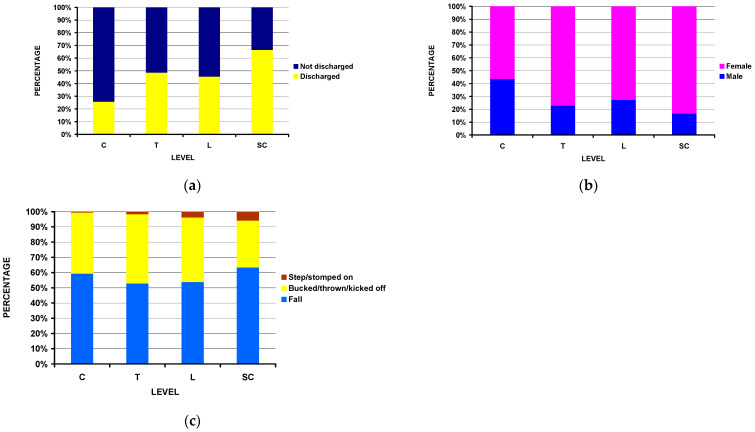
Differences by spinal level, C = cervical, T = thoracic, L = lumbar, and SC = sacrococcygeal: (**a**) by disposition from the ED (*p* < 10^−4^). (**b**) by patient’s sex (*p* = 0.0001). (**c**) by injury mechanism (*p* = 0.031).

There were significant differences by the four major spine levels (Table 2). Hospital admission was highest in the cervical group (74.3%), relatively equal in the thoracic and lumbar groups (51.3% and 58.6%, respectively), and lowest in the sacrococcygeal group (33.5%) (Figure 2a). While females predominated in all the groups (Figure 2b), the cervical group had a higher percentage of males (43.7%) compared to the thoracic, lumbar, and sacrococcygeal groups (22.8%, 27.3%, 16.8%, respectively) (*p* = 0.0001). While a fall off the horse was the predominant injury mechanism in all the groups (Figure 2c), it accounted for 2/3 of the sacrococcygeal group compared to ½ for the cervical, thoracic, and lumbar groups; the lumbar and sacrococcygeal groups also contained a greater percentage of patients that were stepped/stomped on by the horse (*p* = 0.031). 

### 3.3. Differences by Sex

**Figure 3:**
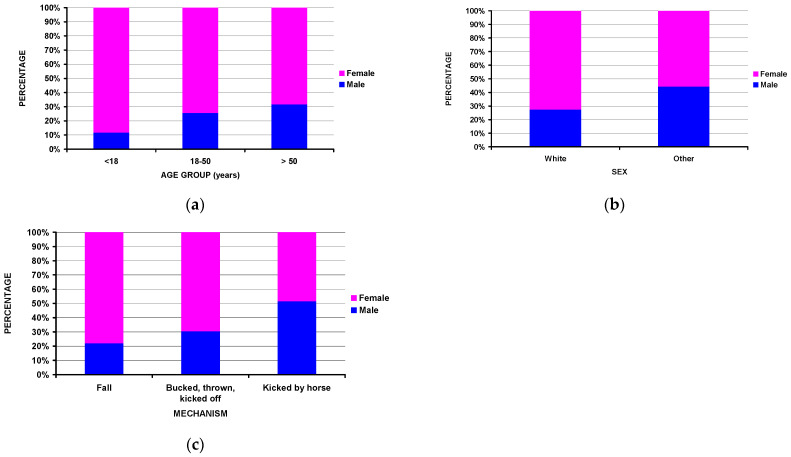
Differences by sex: (**a**) by three major age groups (*p* = 0.008), (**b**) by race (*p* = 0.036), and (**c**) by injury mechanism (*p* = 0.009).

**Figure 4:**
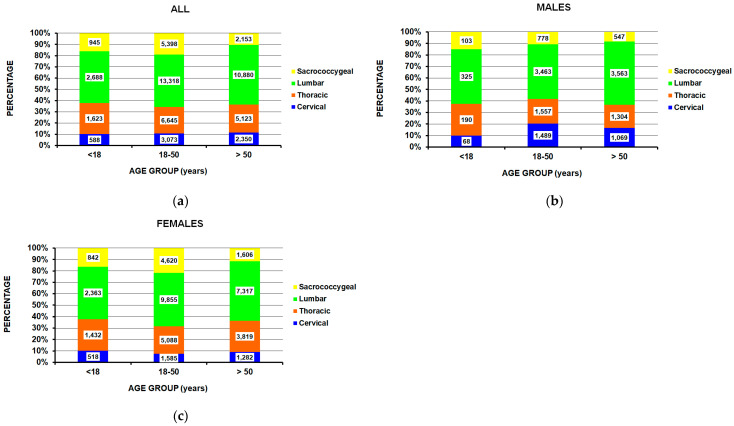
Spine injury location by age group: (**a**) both male and female (*p* = 0.16), (**b**) for males (*p* = 0.73), and (**c**) for females (*p* = 0.097).

Differences by sex were also noted (Table 3). While females comprised the majority across all the ages, there were proportionally fewer females in those over 50 years of age (Figure 3a), where the male percentage was 11.7%, 25.6%, and 31.6% for those <18 years, 18–50 years, and >50 years old, respectively (*p* = 0.008). While the majority of injuries occurred in White individuals, other racial groups were present in 9.8% of the males and 4.9% of the females (*p* = 0.036) (Figure 3b). Of those kicked by the horse, males were equal to female (5l.6% male), while females predominated in those falling off the horse (78.0%) or being bucked off the horse (69.6%) (*p* = 0.0026) (Figure 3c). Further analyses of the major spine location by the three age groups, in total as well as separated by sex, demonstrated no differences (Figure 4).

### 3.4. Associated Injuries

**Figure 5:**
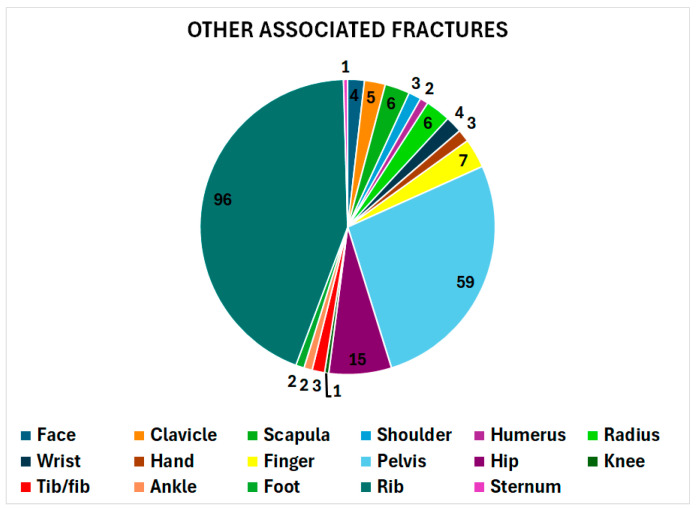
Anatomic location of the 219 associated fractures. The fracture at the 12 o’clock position is the face (4 cases); the pie slices then move clockwise as shown in the legend, with the sternum at 11:59 (1 case), right after the 96 cases of rib(s) fractures.

Using non-weighted data due to the smaller number of cases, there were 306 other injuries in 268 patients (20.7%): 219 were fractures and 87 non-fractures. Of the 219 fractures, 43.8% involved the rib(s) and 26.9% the pelvis, with the remainder shown in Figure 5. The 87 non-fracture injuries were a traumatic brain injury in 37 (43%); lung/pneumo/hemothorax in 29 (33%); spleen/liver in seven (8%); renal/adrenal and mediastinal in three each (3%); intestinal, aortic, and vertebral artery injuries in two each (2%); and vaginal/bladder and tongue injuries in one each (1%).

### 3.5. Illustrative Case Examples from the Narrative Comments

1. A 36-year-old male was riding a horse in a race when the horse stopped, causing him to fall. He was wearing a protective vest. He sustained a loss of consciousness along with fractures of the T3, T4, and T5 vertebrae.
2. A 45-year-old female fell from a horse while jumping, landing on the back of her neck, sustaining a fracture of both the C1 and C2 vertebrae.
3. A 22-year-old female was thrown off a horse while team roping, sustaining transverse process fractures of L2,3,4.
4. A 56-year-old male was thrown from a horse, sustaining a type III odontoid fracture, a burst fracture of T3 and 4, closed fractures of multiple ribs, a mediastinal hematoma, and a tongue laceration.
5. A 73-year-old female fell off a horse, sustaining a left clavicle fracture, rib fractures with hemopneumothorax, thoracic transverse process fractures, and an intimal tear of the aorta.
6. A 55-year-old female was bucked off a horse, coming down hard on the saddle. She sustained a sacral pelvic fracture, open book pelvic fracture, and lacerations of the bladder and vaginal wall.
7. An 87-year-old helmeted male was horse jumping three days ago when he was thrown from the horse, landing on his head. He sustained a type II odontoid fracture.
8. A 39-year-old female was riding her horse after having some drinks at a local bar when the horse became agitated, and she was bucked from the horse, sustaining a sacral fracture.
9. A 38-year-old female fell off a cantering horse and sustained both lumbar and sacral fractures.
10. A 13-year-old male was riding a horse that was struck by a car. The horse died at the scene, and the patient was amnestic to the event, sustaining a cervical spine fracture.
11. A 62-year-old female was climbing onto a horse when she accidentally kicked the ladder; the horse started running, and the patient fell. The patient tested positive for amphetamines and THC. She sustained an L1 burst fracture, sacral hematoma, and traumatic epistaxis.

## 4. Discussion

In this study, there were 54,830 spinal injuries due to equestrian activity, with nearly all being fractures (99.9%); neurologic injury was described in 1.8%. The anatomic distribution of the spine injury was the lumbar spine (49.1%), followed by the thoracic (24.4%), sacrococcygeal (15.5%), and cervical (11.0%) spine. It is difficult to compare our findings to those in the literature, as some did not include the sacrococcygeal spine [11,19,20]; lumped the lumbar and sacrococcygeal spine together [21,34]; or lumped the thoracic, lumbar, and sacral spine into one group in comparison to the cervical spine [4].

When sacrococcygeal fractures are excluded, then the percentages in this study become 13.0% cervical, 28.9% thoracic, and 58.1% lumbar, similar to the 20.9% cervical, 34.3% thoracic, and 44.8% lumbar in the study of Myers et al. [20]. The UK Meyers study involves all sporting activities, with horse riding the most common activity associated with spinal fractures (55 of 122—45%). In a study from Puerto Rico [19], the location was 69% cervical, 9% thoracic, and 22% lumbar. This high percentage of cervical spine injuries is likely due to the fact that it was a study from a neurosurgery database, and cervical spine injuries are often cared for by neurosurgeons instead of orthopedic surgeons in many centers. In that study [19], there was a preponderance of men (87%). According to that study [19], horseback riding in the streets of Puerto Rico is much more common among men, where they participate in a procession termed a “cabalgata”.

Regarding the vertebral level of injury, in this study, 42.1% involved the T12-L2 levels. In a study from the Netherlands of spine fractures due to horse riding [3], 36 fractures occurred in 32 patients, with the majority (78%) occurring at the thoracolumbar junction (T12-L1). They excluded sacrococcygeal fractures; when excluding SC and L2 fractures in our study, the percentage at T12-L1 is 37.6%. The marked difference is likely due to the fact that the Netherlands study involved only patients admitted to the hospital, had only one patient with a cervical fracture, and was a relatively small study of 32 patients. Thus, this study likely gives the best breakdown to date of the anatomic location of spine injuries due to equestrian activity.

In this study the majority were female (73.6%), very similar to the Netherlands study [3], where 88% were female (28 of 32). This is different from the Australian study [21] of 30 vertebral column fractures due to horse riding, where females comprised 53% (16 of 30). In this Australian study [21], men were more often occupational riders (86%), with leisure riders more often women (58%). The high percentage of female injuries is not surprising, as females predominate in equestrian-related activities [26,27,28]. In this study there were more males in the >50 year age group (31.6%), compared to the 11.7% and 25.6% for those <18 years and 18–50 years old. We suspect that this may be due to the males being occupational riders/handlers compared to leisure activity equestrians. Unfortunately, the NEISS does not give information on whether the injury occurred while at work.

While females predominated in all four major locations, cervical injuries had a much higher percentage of males (43.7%) compared to the 22.8%, 27.3%, and 16.8% male for thoracic, lumbar, and sacrococcygeal injuries, respectively. It is possible that cervical injuries are more frequent in racing and jumping equestrian sports, including three-day eventing, show jumping, and racing on the flat and over fences. In racing there is a predominance of male athletes, and in upper-level eventing and show jumping, the proportion of males to females increases versus lower-level/recreational riding. Thus, these sex differences may reflect that injuries are more frequent in higher-level jumping and racing disciplines when speeds, jumps, etc., are all faster and higher, with a greater propensity for cervical spine injuries compared to the other locations.

Using hospital admission as an indicator of injury severity, cervical injuries were the highest (74.3%), with thoracic and lumbar at 51.3% and 58.6%, followed by sacrococcygeal injuries at 33.5%. This is quite understandable, as the potential neurologic risk for discharging a cervical fracture is clearly greater than for the other three spinal segments. The tragic case of Christopher Reeves occurred while jumping during the cross-country phase of a low-level eventing competition. These injuries have the possibility of being catastrophic secondary to spinal cord injury, but in this overall nationwide study, neurologic injury was relatively rare. Similarly, only 1048 of the 54,830 (1.9%) cases in this study occurred while jumping.

The ED encounter occurred most commonly in small hospitals (38.2%), defined by the NEISS as ≤16,830 annual ED visits. Thus, small hospitals should be aware of this fact, especially ED health care providers, as well as administrators who supply resources for the health care providers.

In this study alcohol consumption occurred in 1.7% of the patients. It should go without saying that alcohol or other substance use should not occur while being involved with equines. In a 1992 report, the North Carolina Office of the Chief Medical Examiner [35] described 30 horseback-riding deaths associated with alcohol use, with one to three occurring each year. Of these 30, 16 (53%) were male with a median age of 33.5 years. Twenty-one (70%) riders died when they fell or were thrown from the horse. Twenty (67%) riders died following head injuries (including one rider who drowned after striking his head, losing consciousness, and rolling into water); nine (30%) riders died from internal chest or abdominal injuries; and one rider drowned when he rode his horse into a lake. One died from a spinal hemorrhage.

Ball et al. noted alcohol ingestion in five of 151 equestrian injured patients [36]. In a series of 112 patients [19] with neurosurgical (both cranial and spinal trauma) equestrian-related injuries, one patient was riding while using illicit drugs, and nine patients were intoxicated with alcohol. In 47 patients admitted to a Kentucky level I trauma center for equestrian injuries [37] who had blood alcohol measurements, alcohol was present in 8 (17%). In a questionnaire study of 20 patients injured in horse-riding accidents [22], 4 admitted to alcohol use. Alcohol use likely impairs equestrians by affecting coordination, judgment, and reaction times [38,39,40,41,42,43,44]. The effect of alcohol is also increased in younger individuals [40], and the effects of alcohol and cannabis are additive [38,45].

The attitudes toward horse riding and drinking may be similar to those of cycling. In a German study [46] comparing alcohol use in those driving versus cycling, drunk cycling was seen as more acceptable and less dangerous than driving after alcohol consumption, and perhaps the same applies to horse riders. Persons who cycled more often under the influence observed such activity more often among their friends, perhaps perceiving less of a danger to themselves and others when cycling after alcohol consumption, although they agreed less with the statement that one should leave one’s bike parked after alcohol consumption [46]. Finally, in 22 of the 50 US states, horseback riders can be charged with a DUI (driving under the influence) [47].

### 4.1. Injury Prevention and Protective Equipment

Prevention of an injury is always preferred to treatment. The use of external protective devices for jumping was first recommended in 1994, and “body-protecting vests” in 1996, became mandatory for the cross-country phase of events. United States Pony Club members are now required to don protective vests during cross-country competitions [48]. However, the actual efficacy of such vests in the prevention of thoracic/lumbar fractures is unknown, and likely would have no role in preventing cervical or sacrococcygeal fractures. Vests can be either standard cloth [flak jacket] types [49] or air vests (similar to automobile airbags). Early in the era of protective vests (1992–1997), Whitlock [50] recorded 193 injuries where riders were wearing some form of body protector. There were two patients with spine fractures due to direct impact with the ground during a fall. One case involved a fracture of T12, and the other several transverse process fractures of the lumbar spine. The protective vest worn at the time was designed to reduce soft tissue injury, and possibly provide some protection to the chest and spine from a fall or kick [50]. There were 24 chest injuries with one fatality. Hessler et al. [51] in 2012 studied 31 patients < 18 years old who sustained torso injuries while horseback riding, and compared them to 61 equestrians with injuries not involving the trunk. They found that safety vest use did not reduce the risk of a torso injury, acknowledging that the development of better vests might lead to different results. Protective vests in rodeo athletes [52] do not seem to impact the occurrence of fatal thoracic injuries. However, Meyer et al. [53] found that show jumping riders who often or always wore a safety vest suffered significantly fewer spinal injuries and concluded that it should be a requirement to wear safety vests in show jumping in general. The exact differences in the anatomic location of the spinal injuries between the vest wearers and non-wearers, however, were not delineated in the Meyer [53] study. Given the conflicting reports, it remains controversial whether standard protective vests currently afford protection against spinal injury.

Air vests have been associated with an increased number of high-risk landings in jumping during cross-country events [54,55]. In a systematic review of airbag vests, there was no reduction in injury and may be associated with an increased risk of serious or fatal injuries in certain settings [55]. Nylund et al. [49], in a single study, noted that riders wearing an air jacket had 1.7 times increased odds of sustaining serious or fatal injury in a fall compared to riders not wearing an air jacket. They postulated several possible reasons for this increase: One, the pull force applied to riders may alter their fall trajectory, increasing the risk of landing closer to the horse, and thereby increasing the risk of crush or trampling injury. Secondly, the air jacket airbags inflate along the long axis of the torso, which may restrict the rider’s torso movement, impeding their ability to tuck-and-roll following ground impact. Third, the high sound level (87–98 dB) when the CO_2_ cartridge is activated may momentarily distract the equestrian from responding to the fall. The American Academy of Audiology notes that a 90 dB level is common to lawnmowers, power tools, and blenders, and a 100 dB level is common to snowmobiles [56].

Another prevention strategy is education, especially regarding alcohol use. O’Brien et al. [57] stated that education is the mainstay regarding the appropriate social use of alcohol in athletes, and clearly, equestrians are considered athletes. They state that “Athletes and coaches need to be aware of the sports related adverse effects of alcohol consumption and its role in sports injury and poor physiological performance. It is recommended that alcohol should be avoided by the serious athlete”. Education regarding the positives of preventive equipment should also be given. A study of German equestrians [58] found that attitudes towards safety products, as well as the protective behavior of other horse owners and riding pupils from the stable, are key factors influencing the safety behavior of equestrians. In rodeo athletes, the perception that vest usage was required encouraged the athletes to wear them [59]. For those engaged in jumping, specific rider training may be beneficial, as demonstrated by a video analysis study of equestrian-related falls [54].

### 4.2. Limitations

There are several limitations of this study. First, the study is based on ED data; the type of treatment, ISS scores, and final outcomes are unknown. As it is an ED-based study, patients seen in urgent care centers or private physicians are not captured; however, any severe spine injury is likely seen in the ED. The narrative comments did not always give details regarding the exact vertebral level, and/or the type of neurologic injury sustained. It is also possible that there were other patients with spine fractures due to horse activity, but were not noted in the narrative comments and thus not found. Similarly, the role of jumping and alcohol was dependent upon the narrative comments. However, a spine injury is a significant event, and it is unlikely that it was not included in the narrative comments. Finally, the treatment and final long-term clinical outcomes for these patients are not known. The strength of this study is that it is the largest series of spine injuries due to equestrian activities, and gives health care providers caring for such injuries a broad overview of the problem. Finally, it is baseline data to evaluate the outcomes and/or efficacy of future prevention programs that might be instituted.

## 5. Conclusions

In this study of 54,830 spine injuries due to equine activity, the majority were female (73.6%) and White (93.7%), with a fracture in 99.9%. The anatomic level of the fracture was lumbar (49.1%), thoracic (24.4%), sacrococcygeal (15.5%), and cervical (11.0%); 42.1% occurred at the thoracolumbar junction T12-L2. Cervical injuries had a much higher percentage of males (43.7%). There were multiple-level fractures in 4.0%, and an internal organ injury was present in 5.7%. The majority were seen at small hospitals (38.2%). Neurologic injury was rare (1.8%). Alcohol involvement was present in 1.7% of the patients. This large study can be used as baseline data to evaluate further changes in equestrian injuries, especially the impact of further prevention strategies, education protocols, and legislative/governmental regulations of public equestrian facilities.

## Figures and Tables

**Table 1:** General demographics of equestrian-related spine injuries.

All	n	N	L95%	U95%	%
**Age group (years)**					
**≤10**	17	679	395	1168	1.2
**11 to 17**	117	5165	4375	6081	9.4
**18 to 25**	150	5957	5077	6969	10.9
**26 to 50**	524	22,522	20,876	24,207	41.1
**>50**	486	20,507	18,417	22,689	37.4
					
**≤16**	90	3875	3235	4633	7.1
**>16**	1204	50,955	50,197	51,595	92.9
**Hospital size**					
**Small**	282	20,942	14,475	28,276	38.2
**Medium**	132	9143	5774	13,921	16.7
**Large**	253	15,246	6344	29,137	27.8
**Very large**	575	9197	6092	13,450	16.8
**Children’s**	52	302	170	537	0.6
**Sex**					
**Male**	349	14,455	13,170	15,818	26.4
**Female**	945	40,374	39,012	41,660	73.6
**Race**					
**White**	921	39,692	38,840	40,352	93.7
**Black**	15	621	343	1114	1.5
**Other**	49	2055	1521	2767	4.9
					
**White**	921	39,692	38,840	40,352	93.7
**All others**	64	2677	2017	3529	6.3
**ED disposition**					
**Discharged**	631	26,835	24,992	28,687	48.9
**Not discharged**	663	27,995	26,143	29,838	51.1
**Most proximal level**					
**Cervical**	141	6011	4920	7308	11.0
**Thoracic**	326	13,391	11,949	14,945	24.4
**Lumbar**	731	26,886	25,404	28,373	49.1
**Sacrococcygeal**	195	8496	7352	9779	15.5
**Multiple levels**					
**Yes**	59	2191	1672	2868	4.0
**No**	1235	52,638	51,962	53,158	96.0
**Internal organ injury**					
**Yes**	81	3125	2484	3920	5.7
**No**	1213	51,705	50,910	52,346	94.3
**Tack involved**					
**Yes**	22	831	493	1398	1.5
**No**	1272	53,999	53,432	54,337	98.5
**Horse spooked**					
**Yes**	43	1913	1393	2615	3.5
**No**	1251	52,917	52,215	53,437	96.5
**While jumping**					
**Yes**	22	1048	652	1678	1.9
**No**	1272	53,782	53,152	54,178	98.1
**Mounted on horse**					
**Yes**	1278	53,976	53,470	54,298	98.4
**No**	1	15	0	115	0.0
**Mounting**	7	315	121	817	0.6
**Dismounting**	8	523	258	1058	1.0
**Mechanism**					
**Fall**	706	29,190	26,522	31,824	53.6
**Bucked/thrown/kicked off**	510	21,606	19,538	23,746	39.7
**Step/stomped**	38	1696	1160	2466	3.1
**Kicked**	4	161	44	610	0.3
**Tack involved**	1	17	0	125	0.0
**Motor vehicle crash**	3	203	65	637	0.4
**Bite**	0	0	0	0	0.0
**Struck**	10	528	283	866	1.0
**Other**	17	1038	550	1933	1.9
**Neurologic injury**					
**Yes**	19	962	625	1469	1.8
**No**	1275	53,868	53,361	54,205	98.2
**Alcohol present**					
**Yes**	25	958	521	1755	1.7
**No**	1269	53,872	53,075	54,309	98.3
**Fracture present**					
**Yes**	1282	54,370	54,271	54,391	99.9
**No**	2	33	11	131	0.1

n = actual number; N = estimated number; L95% and U95% = the lower and upper 95% confidence intervals of the estimate.

**Table 2:** Differences by spine level.

Variable	Cervical					Thoracic					Lumbar					Sacrococcygeal					
	**n**	**N**	**L95%**	**U95%**	**%**	**n**	**N**	**L95%**	**U95%**	**%**	**n**	**N**	**L95%**	**U95%**	**%**	**n**	**N**	**L95%**	**U95%**	**%**	***p* Value**
**All**	141	6011	4920	7308	11.0	326	13,391	11,949	14,945	24.4	631	26,886	25,404	28,373	49.1	195	8496	7352	9779	15.5	**-**
**Age (years)**																					
**Average [95% CI]**	43.9 [40.8, 47.0]					42.6 [40.3, 45.0]					43.2 [41.5, 44.9]					38.7 [35.7, 41.6]					<10^−4^
**Median**	48					44					45					39					
**Hospital size**																					
**Small**	35	2584	1771	3465	43.0	64	4732	2989	6824	35.3	141	10,568	7240	14,311	39.3	42	3058	1986	4324	36.0	<10^−4^
**Medium**	17	1175	647	1975	19.5	31	2145	1224	3557	16.0	58	4187	2557	6576	15.6	25	1591	866	2708	18.7	
**Large**	23	1319	565	2599	21.9	66	4057	1671	7632	30.3	122	7354	2984	14,293	27.4	42	2515	1065	4692	29.6	
**Very large**	54	858	539	1319	14.3	147	2357	1492	3573	17.6	293	4678	3049	6923	17.4	81	1305	806	2030	15.4	
**Childrens**	12	74	41	135	1.2	18	100	52	193	0.7	17	99	51	196	0.4	5	28	8	94	0.3	
**Age group (years)**																					
**>16**	131	5537	5091	5777	92.1	294	12,186	11,672	12,558	91.0	598	25,460	24,840	25,899	94.7	180	7726	7239	8036	90.9	0.13
**≤16**	10	474	234	920	7.9	32	1204	833	1719	9.0	33	1426	987	2046	5.3	15	770	460	1257	9.1	
																					
**<18**	12	588	310	1069	9.8	43	1623	1217	2140	12.1	60	2688	2065	3474	10.0	19	945	616	1419	11.1	0.16
**18–50**	64	3073	2534	3608	51.1	156	6645	5571	7723	49.6	327	13,318	12,311	14,325	49.5	126	5398	4627	6095	63.5	
**>50**	65	2350	522	901	39.1	127	5123	4021	6323	38.3	244	10,880	9760	12,040	40.5	50	2153	1556	2884	25.3	
**Sex**																					
**Male**	60	2625	2177	3091	43.7	88	3051	2516	3662	22.8	170	7351	6275	8536	27.3	31	1428	1063	1894	16.8	0.0001
**Female**	81	3386	2920	3834	56.3	238	10,339	9729	10,875	77.2	461	19,535	18,350	20,611	72.7	164	7069	6611	7433	83.2	
**Race**																					
**White**	96	4130	3749	4327	91.8	248	9868	9500	10,069	95.9	433	19,473	18,884	19,892	93.5	144	6221	5633	6516	92.0	0.38
**Other**	7	369	172	750	8.2	11	419	219	788	4.1	34	1350	931	1939	6.5	12	539	251	1127	8.0	
**ED disposition**																					
**Released**	39	1545	1110	2078	25.7	157	6515	5605	7431	48.7	304	13,126	11,988	14,268	48.8	131	5649	4711	6456	66.5	0.0006
**Admitted**	102	4466	3933	4901	74.3	169	6876	5960	7786	51.3	327	15,760	12,618	14,898	58.6	64	2847	2040	3785	33.5	
**Internal organ injury**																					
**Yes**	15	469	276	779	7.8	28	979	640	1477	7.3	30	1272	828	1938	4.7	8	403	178	883	4.7	0.33
**No**	126	5542	5232	5735	92.2	298	12,411	11,914	12,751	92.7	601	25,614	24,948	26,058	95.3	187	8093	7613	8318	95.3	
**Injury mechanism**																					
**Fall**	72	3205	2643	3753	53.3	170	6760	5747	7765	50.5	343	13,894	12,131	15,640	52.0	121	5331	4624	5979	62.7	0.16
**Bucked/thrown/kicked off**	57	2167	1673	2716	36.1	142	5834	4954	6741	43.6	246	10,964	9504	12,486	41.0	64	2596	1923	3383	30.6	0.039 ^
**Step/stomped on**	2	31	8	125	0.5	4	206	86	492	1.5	23	970	561	1654	3.6	9	488	252	920	5.7	0.041 ^#^
**Kicked by horse**	0	0	0	0	0.0	0	0	0	0	0.0	4	161	43	607	0.6	0	0	0	0	0.0	
**Tack malfunction**	0	0	0	0	0.0	0	0	0	0	0.0	1	17	3	126	0.1	0	0	0	0	0.0	
**Motor vehicle crash**	2	136	33	530	2.3	0	0	0	0	0.0	1	67	8	497	0.3	0	0	0	0	0.0	
**Bitten by horse**	0	0	0	0	0.0	0	0	0	0	0.0	0	0	0	0	0.0	0	0	0	0	0.0	
**Struck**	5	248	88	670	4.1	2	126	30	504	0.9	3	154	51	473	0.6	0	0	0	0	0.0	
**Misc**	3	223	73	644	3.7	5	233	93	572	1.7	8	501	214	1155	1.9	1	81	10	592	1.0	
**Mounting status**																					
**On horse**	139	5991	5911	6007	99.7	324	13,309	12,847	13,379	99.4	620	26,215	25,738	26,496	97.5	194	8417	7942	8485	99.1	0.091
**Not on horse**	1	15	2	112	0.2	0	0	0	0	0.0	0	0	0	0	0.0	0	0	0	0	0.0	
**Mounting**	1	5	1	36	0.1	2	83	12	545	0.6	4	228	75	683	0.8	0	0	0	0	0.0	
**Dismounting**	8	313	8	64	5.2	0	0	0	0	0.0	7	444	199	973	1.7	1	79	11	554	0.9	
**Horse spooked**																					
**Yes**	3	36	11	123	0.6	10	412	2049	817	3.1	23	1228	807	1850	4.6	7	236	88	619	2.8	0.011
**No**	138	5975	5888	6000	99.4	316	12,979	12,574	13,186	96.9	608	25,659	25,036	26,079	95.4	188	8260	7877	8408	97.2	
**Jumping**																					
**Yes**	5	210	100	433	3.5	3	150	40	550	1.1	12	657	333	1277	2.4	2	31	4	219	0.4	0.21
**No**	136	5801	5578	5911	96.5	323	13,241	12,841	13,351	98.9	619	26,230	25,609	26,553	97.6	193	8466	8277	8492	99.6	
**Tack involved**																					
**Yes**	1	15	2	108	0.2	6	178	74	427	1.3	10	515	245	1078	1.9	5	123	38	387	1.4	0.069
**No**	140	5997	5903	6009	99.8	320	13,213	12,964	13,317	98.7	621	26,371	25,808	26,641	98.1	190	8373	8109	8458	98.6	

n = actual number; N = estimated number; L95% and U95% = the lower and upper 95% confidence intervals of the estimate. ^ *p* value for fall, bucked/thrown/kicked off, and step/stomped on. ^#^ *p* value for fall, and bucked/thrown/kicked off.

**Table 3:** Differences by sex.

Variable	Male					Female					
	n	N	L95%	U95%	%	n	N	L95%	U95%	%	*p* Value
**All**	349	14,455	13,175	15,840	26.4	945	40,374	39,011	41,659	73.6	-
**Age (years)**											
**Average [95% CI]**	45.8 [43.8, 47.8]					41.2 [39.4, 42.9]					0.021
**Median [quartiles]**	46 [32, 58]					43 [26, 54]					
**Hospital size**											
**Small**	78	5757	3322	4701	39.8	204	15,185	27,244	31,096	37.6	0.57
**Medium**	33	2268	2716	4624	15.7	99	6875	27,458	32,788	17.0	
**Large**	63	3788	2971	4295	26.2	190	11,458	28,379	32,077	28.4	
**Very large**	159	2550	3510	4546	17.6	416	6648	27,676	30,571	16.5	
**Childrens**	16	93	52	163	0.6	36	209	24,285	31,128	0.5	
**Age group (years)**											
**>16**	334	13,932	3595	4316	96.4	870	37,023	28,278	30,333	91.7	0.0042
**≤16**	15	523	1120	3254	3.6	75	3351	31,286	37,245	8.3	
											
**<18**	20	686	413	1125	4.7	114	5158	4304	6149	12.8	0.008
**18–50**	168	7286	6466	8105	50.4	506	21,193	19,440	22,928	52.5	
**>50**	161	6483	5663	5392	44.8	325	14,024	12,100	25,314	34.7	
**Race**											
**White**	259	10,868	2977	3644	90.2	662	28,825	28,200	29,270	95.1	0.036
**Other**	30	1185	3874	6885	9.8	34	1492	1046	2116	4.9	
**ED disposition**											
**Released**	161	7003	6275	7735	48.4	470	19,832	18,269	21,398	49.1	0.82
**Admitted**	188	7453	6720	8180	51.6	475	20,542	18,976	22,105	50.9	
**Internal organ injury**											
**Yes**	33	1247	864	1775	8.6	48	1878	1348	2596	4.7	0.042
**No**	316	13,209	12,680	13,591	91.4	897	38,497	37,778	39,026	95.4	
**Injury mechanism**											
**Fall**	162	6418	5675	7178	44.4	544	22,772	20,407	25,061	57.0	0.0009
**Bucked/thrown/kicked off**	157	6569	5762	7394	45.4	353	15,037	13,246	16,916	37.6	0.0009 ^
**Step/stomped on**	18	756	434	1295	5.2	20	940	532	1647	2.4	0.0016 ^#^
**Kicked by horse**	2	83	22	321	0.6	2	78	20	304	0.2	
**Tack malfunction**	1	17	3	126	0.1	0	0	0	0	0.0	
**Motor vehicle crash**	2	135	33	538	0.9	1	68	4	216	0.2	
**Bitten by horse**	0	0	0	0	0.0	0	0	0	0	0.0	
**Struck**	1	38	6	283	0.3	9	489	248	956	1.2	
**Misc**	6	440	214	890	3.0	11	598	284	1243	1.5	
**Mounting status**											
**On horse**	345	14,246	13,920	14,374	98.6	933	39,730	39,296	39,990	98.4	0.099
**Not on horse**	0	0	0	0	0.0	1	15	4	113	0.0	
**Mounting**	1	5	0	36	0.0	6	311	117	807	0.8	
**Dismounting**	3	205	77	533	1.4	5	319	145	703	0.8	
**Horse spooked**											
**Yes**	6	198	71	539	1.4	37	1715	1252	2342	4.2	0.0001
**No**	343	14,258	13,916	14,384	98.6	908	38,659	38,032	39,122	95.8	
**Jumping**											
**Yes**	5	168	75	371	1.2	17	879	4	541	2.2	0.09
**No**	344	14,287	14,084	14,380	98.8	928	39,495	38,949	39,833	97.8	
**Tack involved**											
**Yes**	8	314	143	676	2.2	14	517	246	1078	1.3	0.37
**No**	341	14,141	13,779	14,312	97.8	931	39,857	39,296	40,128	98.7	

n = actual number; N = estimated number; L95% and U95% = the lower and upper 95% confidence intervals of the estimate. ^ *p* value for fall, bucked/thrown/kicked off, and step/stomped on. ^#^ *p* value for fall, and bucked/thrown/kicked off.

## Data Availability

The data is in the public domain and available at the NEISS website.
